# Nuclear expression of Rac1 in cervical premalignant lesions and cervical cancer cells

**DOI:** 10.1186/1471-2407-12-116

**Published:** 2012-03-23

**Authors:** Miguel A Mendoza-Catalán, Gema R Cristóbal-Mondragón, Jesús Adame-Gómez, Heidi N del Valle-Flores, José Fco Coppe, Laura Sierra-López, Mirna A Romero-Hernández, Luz del Carmen Alarcón-Romero, Berenice Illades-Aguiar, Eduardo Castañeda-Saucedo

**Affiliations:** 1 Laboratorio de Biología Celular del Cáncer, UACQB, Universidad Autónoma de Guerrero, Guerrero, Mexico; 2 Unidad de Patología. Hospital Vicente Guerrero, IMSS, Acapulco, Guerrero, Mexico; 3 Laboratorio de Citopatología, UACQB, Guerrero, Mexico; 4 Laboratorio de Biomedicina Molecular, UACQB, Guerrero, Mexico; 5 Laboratorio de Biología Celular del Cáncer. Edificio "F" segundo piso, UACQB, Universidad Autónoma de Guerrero. Ciudad Universitaria, Av. Lázaro Cárdenas s/n, Chilpancingo, Guerrero CP. 39090, Mexico

**Keywords:** Rho-GTPases, Carcinogenesis, Risk factors, Rac1

## Abstract

**Background:**

Abnormal expression of Rho-GTPases has been reported in several human cancers. However, the expression of these proteins in cervical cancer has been poorly investigated. In this study we analyzed the expression of the GTPases Rac1, RhoA, Cdc42, and the Rho-GEFs, Tiam1 and beta-Pix, in cervical pre-malignant lesions and cervical cancer cell lines.

**Methods:**

Protein expression was analyzed by immunochemistry on 102 cervical paraffin-embedded biopsies: 20 without Squamous Intraepithelial Lesions (SIL), 51 Low- grade SIL, and 31 High-grade SIL; and in cervical cancer cell lines C33A and SiHa, and non-tumorigenic HaCat cells. Nuclear localization of Rac1 in HaCat, C33A and SiHa cells was assessed by cellular fractionation and Western blotting, in the presence or not of a chemical Rac1 inhibitor (NSC23766).

**Results:**

Immunoreacivity for Rac1, RhoA, Tiam1 and beta-Pix was stronger in L-SIL and H-SIL, compared to samples without SIL, and it was significantly associated with the histological diagnosis. Nuclear expression of Rac1 was observed in 52.9% L-SIL and 48.4% H-SIL, but not in samples without SIL. Rac1 was found in the nucleus of C33A and SiHa cells but not in HaCat cells. Chemical inhibition of Rac1 resulted in reduced cell proliferation in HaCat, C33A and SiHa cells.

**Conclusion:**

Rac1 is expressed in the nucleus of epithelial cells in SILs and cervical cancer cell lines, and chemical inhibition of Rac1 reduces cellular proliferation. Further studies are needed to better understand the role of Rho-GTPases in cervical cancer progression.

## Background

Cervical cancer is the second most common malignant neoplasia affecting woman worldwide. Infection with High-Risk Human Papillomavirus (HR-HPV) is considered the main risk factor for developing cervical cancer and its precursor lesions [1-3]. Development of cervical Low-grade Squamous Intraepithelial Lesions (L- SIL) and High-grade Squamous Intraepithelial Lesions (H-SIL), and progression to invasive carcinoma, are associated with alterations in the regulation of several cellular processes such as cell cycle progression, apoptosis, and DNA repair [2,4,5]. The HR-HPV oncoproteins E6 and E7 are responsible for many of these alterations, they act by binding to, and/or modifying the expression/activity of a growing number of cellular proteins [6], including p53 [7], pRb [8], p21 [9,10], and p27 [11,12]. Rho-GTPases are small signaling proteins involved in the regulation of crucial cellular functions such as cell shape, cell-cell adhesion, cell proliferation, cell division, migration and invasion [13-15]. Experiments using cell culture and animal models have demonstrated an important role for these proteins in carcinogenesis [16,17]. Moreover, it has been shown that expression of some Rho-GTPases and their regulatory proteins is altered in human cancers such as prostate, colon, lung, and breast cancer [18]. Cell culture experiments showed that RhoC regulates invasion and motility of cervical cancer cells [19,20]. Furthermore, it has been reported that RhoC is overexpressed in biopsies from squamous carcinoma of the cervix (SCC) and cervical intraepithelial neoplasia (CIN) II/III when compared to normal cervical epithelium and CIN I [21]. However, expression of other Rho-GTPases has not been investigated in cervical cancer or its precursor lesions. The aim of this study was to investigate the alterations on the expression of the GTPases Rac1, RhoA, and Cdc42, and the Rho GEFs Tiam1 and beta-Pix in cervical premalignant lesions.

## Materials and methods

### Sample selection

102 paraffin-embedded cervical tissue specimens were obtained from the Department of Pathology at the "Vicente Guerrero" General Regional Hospital (IMSS), in Acapulco, Mexico. Eighty-two samples corresponded to cervical biopsies or cones with confirmed histological diagnosis of L-SIL (n = 51) or H-SIL (n = 31), and 20 corresponded to cervical tissue specimens without SIL, selected from patients undergoing hysterectomy for benign conditions, without a history of SIL or abnormal Pap results. One pathologist (LSL) reviewed all of the slides to confirm the diagnoses. All SIL cases were additionally reviewed by a second pathologist (JFC) to establish a consensus diagnosis (discrepancies relative to the original diagnoses were resolved by the interpretation of a third pathologist).

Approval to conduct this study was obtained from the Institutional Ethics Comitee at the "Universidad Autónoma de Guerrero". The study was conducted in compliance with the Helsinki Declaration.

#### Detection of HR-HPV

The presence of HR-HPV was determined by *in situ *hybridization using the GenPoint tyramide amplification signal kit (DAKO, Carpinteria, CA). Briefly, 3- micron paraffin sections were placed on silanized slides, deparaffinized, and incubated for 5 min at 37°C with proteinase K. Samples were dehydrated, and a mixture containing a pool of biotinilated DNA probes (directed against HPV 16, 18, 31, 33, 39, 45, 51, 52, 56, 58, 59 and 68 types) was added to each section. Sections were covered with a glass coverslip, denatured for 10 min at 95°C, and hybridization was performed for 20 h at 37°C in a humidified atmosphere in a Dako hybridizer (Dako, Carpinteria, CA). The slides were incubated with a streptavidin peroxidase-conjugated primary antibody, followed by incubation with biotil-tyramide, and with streptavidin. The reaction was developed by adding DAB, followed by staining with Mayer's hematoxylin (Merck, Germany), and mounted with Entellan mounting medium (Merck, Germany). The positive reaction was seen as a maroon or brown nuclear signal (Additional file 1: Figure S1).

### Immunohistochemistry and immunocytochemistry

For immunohistochemistry, 3-micron paraffin sections were deparaffinized and rehydrated, followed by 20 min incubation in sodium citrate buffer (pH 6.0) at 110°C for antigen retrieval, using a pressure cooker (T-FAL Clipso). Samples were incubated for 10 min with immunodetector peroxidase block solution (Bio-SB Inc. Santa Barbara, CA.) to inactivate endogenous peroxidase, blocked with PBS + 1% BSA during 30 min, and incubated with primary antibodies for 1 h at room temperature. For immunocytochemistry, 5 × 10^4 ^HaCat, C33A, or SiHa cells were plated on glass coverslips in 6-well culture plates. Cells were maintained on DMEM medium (Invitrogen, Carlsbad, CA,) supplemented with 10% FBS (Byproductos, Mexico) at 37°C in a 5% CO_2 _atmosphere. Where indicated, cells were treated with the Rac1 chemical inhibitor NSC23766 (Santa Cruz Biotechnology Inc, CA) at 25 μM or 50 μM. 24 h or 48 h after plating, cells were fixed with methanol-acetone (1:1) for 30 min, washed with PBS and antigen retrieval, blocking and primary antibody incubation were performed as described for immunohistochemistry. Primary antibodies were detected using a Mouse/Rabbit Immunodetector HRPw/DAB kit (Bio-SB Inc. Santa Barbara, CA.), following manufacturer's instructions, samples were counterstained with Harris's hematoxylin and mounted using Entellan mounting medium (Merck, North America Inc). Antibodies used were: Rac1 (C-14), RhoA (C-15), Cdc42 (B-8), Tiam1 (C-16), and beta- Pix (C-19) (Santa Cruz Biotechnology Inc, CA). The intensity of cytoplasmic staining was scored as weak, moderate or strong at 40× magnification (Additional file 2: Figure S2).

#### Cellular fractionation and western blotting

Cells were seeded on petri dishes and incubated for 24 h in the presence or absence of the Rac1 inhibitor NSC23766. Cells were washed with PBS and lysed in 500 μl of buffer A (10 mM HEPES, pH 9.7; 10 mM KCl, 0.1 M EDTA, 1 mM DTT; 0.5 mM PMSF plus protease inhibitors) directly on the plate and the protein lysate was transferred to a new microtube and centrifuged at 15 000 g for 3 min at 4°C. The cytoplasmic fraction (supernatant) was recovered in a new microtube and the pellet was resuspended in 150 μl of RIPA buffer (150 mM NaCl, 1% Triton X-100, 0.5% sodium deoxycholate, 0.1% SDS, 50 mM Tris pH8). The supernatant (nuclear fraction) was transferred to a new microtube. For total protein extracts, cells were lysed with RIPA buffer (50 mM Tris-HCl pH 7.6, 160 mM NaCl, 0.5 mM EDTA/EGTA, 1% Triton X-100, 10% glycerol, 1 mM PMSF and 1 μg/ml leupeptin). Whole cell, cytoplasmic and nuclear proteins were separated by SDS- PAGE in 10% acrylamide gels, transferred to PVDF membranes and detected by Western blot using antibodies against alpha-tubulin, lamin B, Rac1 (all from Millipore) and Tiam1 (Santa Cruz Biotechnology).

#### Cell proliferation assay

8 × 10^3 ^cells were plated on 24-well plates (Sarstedt AG & CO, Germany) and cultured in DMEM medium supplemented with 10% FBS for 24 h. Cells were treated with the Rac1 inhibitor NSC23766 (Santa Cruz Biotechnology Inc, CA) at 25 μM or 50 μM or with vehicle. Cells were fixed after 48 h of treatment, in 4% formaldehyde for 30 min. Cell proliferation was determined using crystal violet assay. The relative number of cells was determined by measuring the optical density of each well at 600 nm in a biophotometer (Eppendorf RS-2312 DH 8.5 mm).

### Statistical analysis

Association between variables was evaluated by Chi squared test or Fisher's exact test, as appropriate. Differences between data were determinate by two-way ANOVA test. A result was considered to be statistically significant when the *p *value was < 0.05. Statistical analysis was performed using the software STATA *v9.2 or GraphPad Prism v5.03*.

## Results

### Overexpression of rho-GTPases and RhoGEFs in cervical pre-malignant lesions

**Figure 1:**
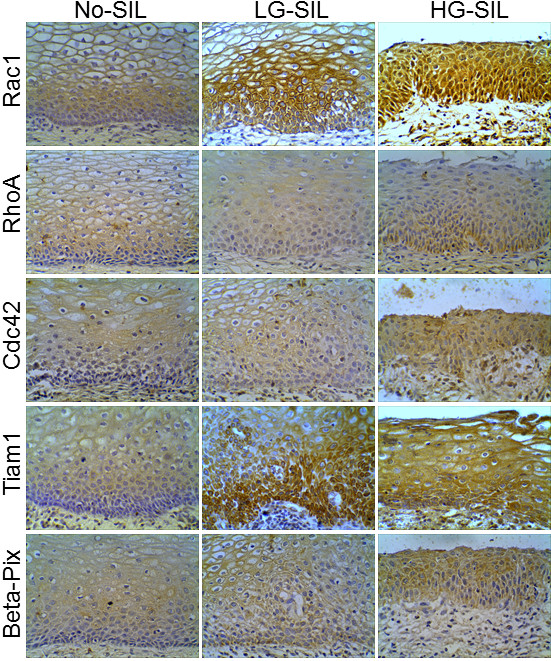
**Expression of Rho-GTPases and Rho-GEFs in cervical epithelium without squamous intraepithelial lesions, low-grade squamous intraepithelial lesions (L-SIL), and high-grade squamous intraepithelial lesions (H-SIL)**. Representative images of immunohistochemical analysis of Rac1, RhoA, Cdc42, Tiam, and beta-Pix expression. Magnification: 40 ×.

We first determined HR-HPV infection as described under materials and methods. 20 samples were negative and 62 were positive for HR-HPV. In 20 samples HR-HPV infection could not be determined (Additional file 3: Table S1). Next, we determined the expression of Rac1, RhoA, Cdc42, Tiam1 and beta -Pix in cervical samples. Expression of the five proteins was observed in all cervical samples and the intensity of the signal for Rac1, RhoA, Tiam1, and beta-Pix was stronger in L-SIL and H-SIL, when compared to samples without SIL (Figure 1). As shown in Table 1, in the majority of samples without SIL the immunoreactivity for the five proteins was weak, only 35% and 20% of these samples had moderate/strong signal for Rac1 and Tiam1, respectively. In contrast, 64.7% L-SIL and 74.2% H-SIL showed moderate/strong signal for Rac1, and 80.4% L-SIL and 80.6% H-SIL had moderate/strong reactivity for Tiam1. Similarly, moderate/strong reactivity of RhoA was observed in 40%, 51%, and 71% for samples without SIL, L-SIL and H-SIL, respectively. Cdc42 reactivity was moderate/strong in 40% of samples without SIL, 41.2% L-SIL, and 61.3% H-SIL. For beta-Pix, moderate/strong reactivity was observed in 25% of samples without SIL, 37.2% L-SIL, and 64.5% H-SIL (Table 1). A significant association was found between the immunoreactivity of Rac1 and L-SIL (*p *= 0.02) and H-SIL (*p *= 0.005); RhoA and H-SIL (*p *= 0.03); Tiam1 and L-SIL (*p *< 0.001) and H-SIL (*p *< 0.001); and beta-Pix and H-SIL (*p *= 0.006). No significant association was found between the reactivity of Cdc42 and L-SIL or H-SIL, or between the reactivity of RhoA or beta-Pix and L-SIL. We found that the intensity of Tiam1 immunoreactivity was associated with HR-HPV infection (*p *= 0.014), whereas no significant association was found between the immunoreactivity of Rac1, Cdc42, RhoA or beta-Pix, and HR-HPV infection (data not shown).

**Table 1:** Association between the intensity of Rac1, RhoA, Cdc42, Tiam1 and beta-Pix immunoreactivity, and the histological diagnosis

		Histological diagnosis			
IR* intensity	**without SIL** **% (n)**	**L-SIL** **% (n)**	**p value†**	**H-SIL** **% (n)**	**p value†**

**Rac1**					

Low	65 (13)	35.3 (18)	**0.02**	25.8 (8)	**0.005**

moderate/high	35 (7)	64.7 (33)		74.2 (23)	

**Cdc42**					

Low	60 (12)	58.8 (30)	0.93	38.7 (12)	0.12

moderate/high	40 (8)	41.2 (21)		61.3 (19)	

**RhoA**					

Low	60 (12)	49 (25)	0.41	29 (9)	**0.03**

moderate/high	40 (8)	51(26)		71(22)	

**Tiam1**					

Low	80 (16)	19.6 (10)	**< 0.001**	19.4 (6)	**< 0.001**

moderate/high	20 (4)	80.4 (41)		80.6 (25)	

**Beta-Pix**					

Low	75 (15	62.8 (32)	0.33	35.5 (11)	**0.006**

moderate/high	25 (5)	37.2 (19)		64.5 (20)	

***Total***	***20***	***51***		***31***	

*IR = immunoreactivity; † = Chi squared test

### Nuclear expression of Rac1 in SILs and cervical cancer derived cell lines

**Figure 2:**
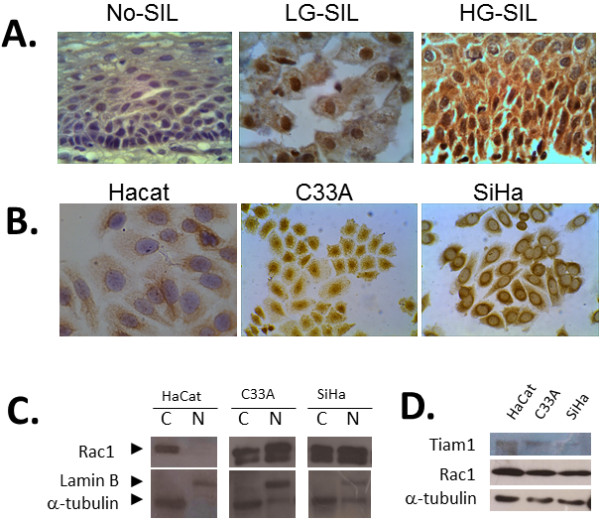
**Nuclear expression of Rac1 in squamous intraepithelial lesions and cervical cancer cell lines**. **A**. Representative images showing nuclear Rac1 expression in low-grade squamous intraepithelial lesions (L-SIL) and high-grade squamous intraepithelial lesions (H-SIL) but not in epithelium without squamous SIL. B. Representative images of immunocytochemical analysis showing nuclear Rac1 expression in cervical cancer cells C33A and SiHa, but not in non-tumorigenic Hacat cells. Magnification: 40×. C. Western blot analysis of Rac1 protein levels in cytoplasmic (C) and nuclear (N) protein extracts from HaCat, C33A and SiHa cells. D. Western blot analysis of Rac1 and Tiam1 protein levels in whole-cell extracts from HaCat, C33A and SiHa cells.

We observed a strong nuclear reactivity for Rac1 in a subset of L-SIL and H-SIL samples, whereas no nuclear reactivity was observed in samples without SIL (Figure 2A). Nuclear staining was observed along all layers of the epithelium and was found in 52.9% L-SIL and 48.4% H-SIL (Table 2). In line with these observations, nuclear immunoreactivity for Rac1 was observed in cervical cancer derived cell lines C33A (HPV-negative) and SiHa (HPV-16), but not in immortalized non-tumorigenic keratinocytes (HaCat). A strong perinuclear Rac1 immunoreactivity was also observed in SiHa cells (Figure 2B). These observations were further confirmed using cellular fractionation and Western blot analyses. As shown in Figure 2D, Rac1 was detected in the cytoplasmic fraction of the three cell lines, and in the nuclear fraction of C33A and SiHa cells, but not in the nuclear fraction of Hacat cells (Figure 2C). Using Western blot analysis on whole-cell extracts from HaCat, C33A and SiHa cells, we found that Rac1 protein levels are similar in all cell lines (Figure 2D).

**Table 2:** Association between the Rac1 nuclear immunoreactivity, and the histopathological diagnosis

		Histopathological diagnosis		
**Rac1 nuclear**	**Without SIL** **% (n)**	**L-SIL** **% (n)**	**H-SIL** **% (n)**	p value*

Negative	100 (20)	47.1 (24)	51.6 (16)	
	
Positive	0 (0)	52.9 (27)	48.4 (15)	< 0.001
	
***Total***	***20***	***51***	***31***	

* = Fisher's exact test: without SIL versus L-SIL, and without SIL versus H-SIL.

### Chemical inactivation of Rac1 reduces its nuclear immunoreactivity and inhibits cell proliferation

**Figure 3:**
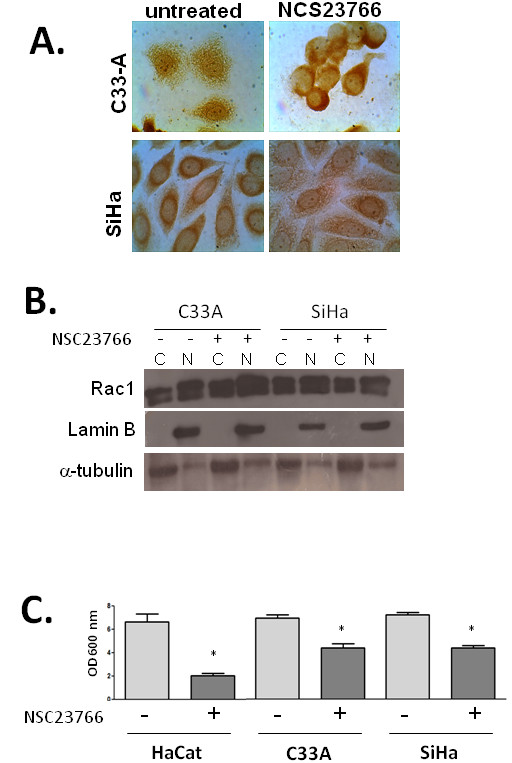
**Effect of the Rac1 chemical inhibitor NSC23677 on the subcellular localization of Rac1, and on cell proliferation**. **A**. Representative images of immunocytochemical analysis for Rac1 in C33A and SiHa cells, treated or not with the Rac1 inhibitor NSC23677. **B**. Detection of Rac1 in cytoplasmic (**C**) and nuclear (N) proteins from HaCat, C33A and SiHa cells treated or not with the Rac1 inhibitor NSC23677. Lamin B was used as a nuclear marker and α-tubilin is a cytoplasmic marker. **C**. Results from crystal violet cell proliferation assays on HaCat, C33A and SiHa cells treated or not with the Rac1 inhibitor NSC23766 for 48 h. Data shown are average optical density values plus standard deviation of experiments performed in triplicate.

To test whether nuclear Rac1 expression in C33A and SiHa cells is dependent on its activation status, C33A and SiHa cells were treated with 25 μM or 50 μM of the Rac1 inhibitor NSC23766. Imnunocytochemical analyses of NSC23766-treated cells showed an apparent reduction in the nuclear Rac1 immunoreactivity in both cell lines, as well as a reduction of the perinuclear immunoreactivity in SiHa cells (Figure 3A). However, cellular fractionation and Western blot analysis demonstrated that treatment with the Rac1 inhibitor does not affect the nuclear localizacion of Rac1 in these cell lines (Figure 3B). We next tested whether the chemical inhibition of Rac1 has an effect on the proliferation of Hacat, C33A and SiHa cells. We found that tNSC23766 treatment resulted in a significant decrease in the proliferation of the three cell lines (Figure 3C).

## Discussion

Overexpression of Rho-GTPases and Rho-GEFs has been described in various types of human tumors [18], and in some cases overexpression is associated with tumor progression or poor prognosis [22,23]. However, little is known about the role of Rho-GTPases in cervical carcinogenesis. Here, using immunohistochemistry, we show that the immunoreactivity of the GTPases Rac1 and RhoA, and the Rho GEFs Tiam1 and beta-Pix, is increased in SILs, compared to cervical epithelium without SIL. Interestingly, we found that Rac1 is present in the nucleus of a subset of L-SIL and H-SIL, but not in samples without SIL. In agreement with these findings, we observed nuclear localization of Rac1 in cancer derived C33A and SiHa cells but not in non-tumorigenic HaCat cells.

Rac1 has a nuclear localization signal (NLS) [24], and it has been recently shown that the importin Karyopherin alpha 2 (KPNA2) mediates Rac1 nuclear import trough the interaction with its NLS, and that KPNA2-mediated nuclear import of Rac1 requires Rac1 activation [25]. Here we show that the nuclear localization of Rac1 in C33A and SiHa cells is not affected by treatment with the Rac1 inhibitor NSC23766. These data indicate that in these cells, the presence of Rac1 in the nucleus is not dependent on its activation. Michaelson et al. (2010) showed that Rac1 translocates to the nucleus during the G2 phase of the cell cycle, and that targeting an active form of Rac1 to the nucleus promotes cell proliferation [26]. We found that chemical inhibition of Rac1 reduces the proliferation of cervical cancer cell lines C33A and SiHa, as well as that of non-tumorigenic HaCat cells. In HaCat cells, in which Rac1 is localized to the cytoplasm, chemical inhibition of Rac1 may reduce its nuclear translocation during the G2 phase of the cell cycle, resulting in a reduction in cell proliferation. Buongiorno et al. (2008) showed that an inactive form of Rac1 is present in the nucleus of colorectal cancer cells, where it associates with the transcription factor TCF-4 [27]. Interestingly, these authors demonstrated that activation of the Wnt signaling pathway induced the nuclear translocation of Tiam1, a Rac1-specific activator, in a complex with beta-catenin, and that once in the nucleus a beta-catenin/Tiam1/TCF4/Rac1 complex can be formed, resulting in the activation of Rac1 and transcriptional activation of Wnt target genes [27]. Activation of the Wnt signaling pathway plays an important role during cervical cancer progression [28,29]; therefore nuclear Rac1 may cooperate with this pathway to stimulate proliferation of cervical cancer cells. We found that chemical inhibition Rac1 in C33A and SiHa cells, in which Rac1 localizes both to the cytoplasm and the nucleus, impairs proliferation without affecting Rac1 nuclear localization. In these cells, inactivation of the nuclear pool of Rac1 may impair the interaction of Rac1 with nuclear proteins such as TCF4 and beta-catenin, resulting in a reduction in the expression of proliferation-related genes and therefore the reduction in cell proliferation. However, Rac1 can also regulate proliferation trough the activation of cytoplasmic signaling pathways such as NF-kB [30], MAPK [31], Jak/Stat [32] and Wnt [33] pathways. Therefore, it is possible that inhibition of the cytoplasmic pool of Rac1 in both cervical cancer-derived and non-tumorigenic cells may result in a reduction of cell proliferation, independently of Rac1 nuclear functions. Altogether, these data suggest that nuclear Rac1 may play an important role in regulating cell proliferation and gene expression in cervical cells, and that the presence of Rac1 in the nucleus of cervical epithelial cells from pre-malignant lesions may contribute to cancer progression.

In our study, we observed overexpression of Rac1, RhoA and Tiam1 in L-SIL and H-SIL, and beta-Pix in H-SIL, when compared with epithelia without SIL. In vitro experiments in HeLa cells demonstrate that Rac1 [34] and Rho [35] activation is required for cell growth and migration. Similarly, experiments in CaSki cells showed that inhibition of migration and invasion by the anticancer agent JOTO1007, is associated with a reduction in the expression of RhoA and the Rho downstream effector ROCK-1 [36]. Moreover, experimental evidences indicate that Rho GTPases play a role in cellular transformation. It has been shown that Rac1 and its activator Tiam1 are required for Src-induced transformation [37]. Similarly, it has been demonstrated that Rac1 and Cdc42 are necessary for H-Ras-induced transformation, although overexpression of constitutively active forms of Rac1 or Cdc42 is not sufficient for cellular transformation [38]. It has also been shown that RhoA overexpression can induce pre-neoplastic transformation of primary mammary epithelial cells [39]. These data suggest that overexpression of Rho GTPases in SILs may cooperate with other signaling pathways to promote tumor progression.

We found that the increased immunoreactivity of Rac1, RhoA and beta-Pix correlates with the histological diagnosis but not with HR-HPV infection. In contrast, Tiam1 immunoreactivity was associated with both histological diagnosis and HR-HPV infection. These observations suggest that altered expression of Tiam1, but not that of Rac1, RhoA and beta-Pix may be dependent of HR-HPV infection. However, further studies are needed in order to determine if increase levels of Rho proteins and their GEFs is induced directly by HPV oncoproteins or is the result of a secondary event related to the progression of the malignancy. Our data indicate that nuclear expression of Rac1 in cervical lesions may be independent of HR-HPV infection as not all HR-HPV positive samples have nuclear staining for Rac1. Moreover, both HPV-negative and HPV-positive cervical cancer derived cells have nuclear staining for Rac1. However, as mentioned above, it is possible that infection with other HPV types not detected by ISH technique we used in this work may affect the subcellular localization of Rac1. Moreover, ISH does not allow us to identify which HR-HPV type is present in the samples, and it is possible that infection with some HR-HPVs such as HVP16 and HPV18 will have a more dramatic effect on the expression of these proteins. This could be of particular relevance for our study population, as in a recent study performed on women from Guerrero state in the south of Mexico, Illades-Aguiar et al., (2010) reported that whereas HPV16 is the most frequent HPV type present in women with cervical cancer, the most frequent type in women with L-SIL was HPV33 [40]. We also found moderate-strong reactivity for the five proteins in samples without SIL (Table 1). Recent evidences demonstrate infection with HR-HPVs in patients without SILs [40-42]. It is possible that some of the samples without SIL that showed moderate-strong reactivity are positive to HR-HPV. As mentioned above, we used ISH for the detection of HR-HPV infection. However this method has limitations as it detects only a subset of HR-HPV types. Further studies using more sensitive techniques such as PCR-RFLP or sequencing for the detection and typing of HPV infection will be required to answer to this concern. Finally, we could not determine HR-HPV infection in a subset of samples. Further investigation is required to determine the possible association between the overexpression of Rho-GTPases and HR-HPV infection.

One of the limitations in our study is that expression of the analyzed proteins in cervical biopsies was studied only by immunochemistry. Further studies using Western blotting, as well as analysis of a larger number of samples are required.

## Conclusions

In conclusion, Rac1 and Tiam1 are overexpressed in L-SIL and H-SIL, RhoA and beta-Pix are overexpressed in H-SIL. Rac1 is expressed in the nucleus of cervical premalignant-lesions and cervical cancer derived cells lines. The chemical inhibition of Rac1 inhibits cell proliferation in Hacat, C33A and SiHa cells. To our knowledge, this is the first report showing abnormal expression of Rho-GTPases in cervical cancer, Further studies are needed to better understand the role of the overexpression of Rho-GTPases, as well as the nuclear Rac1 expression in cancer progression.

## Competing interests

The authors declare that they have no competing interests.

## Authors' contributions

MAMC, GRCM, JAG and HNVF performed the experimental procedures and analyzed the data; MAMC performed statistical analyses; LSL and JFC performed the histopatological diagnosis and participated in the interpretation of IHC results; LSL and MAR, participated in sample selection and review of patient's files. MAR and LCAR performed detection of HR-HPV. BIA and MAMC participated in interpretation and analysis of data and manuscript preparation. ECS conceived the project, designed experiments, analyzed the data, supervised the whole project and wrote the manuscript. All authors read and approved the final version of the manuscript.

## Pre-publication history

The pre-publication history for this paper can be accessed here:

http://www.biomedcentral.com/1471-2407/12/116/prepub

## Supplementary Material

Additional file 1 **Figure S1**. HR-HPV detection by ISH. Representative images of (a) L-SIL sample in which HPV-probe set was excluded (negative control); (b-d) HR-HPV positive H-SIL showing strong nuclear staining. (a-c) 40×, (d) 100 ×. Click here for file

Additional file 2 **Figure S2**. Establishment of the criteria for interpretation of IHC results. To analyze the differences in the intensity in immunoreactivity of the five proteins, we defined four categories based on signal intensity: a) negative, b) low, c) moderate and d) strong. Click here for file

Additional file 3 **Table S1**. Characteristics of the study population. Click here for file
