# Supplementary figures and images for: Nuclear expression of Rac1 in cervical premalignant lesions and cervical cancer cells
Source: BMC Cancer. 2012 Mar 23;12:116. doi: 10.1186/1471-2407-12-116 (PMC3340301; doi:10.1186/1471-2407-12-116)

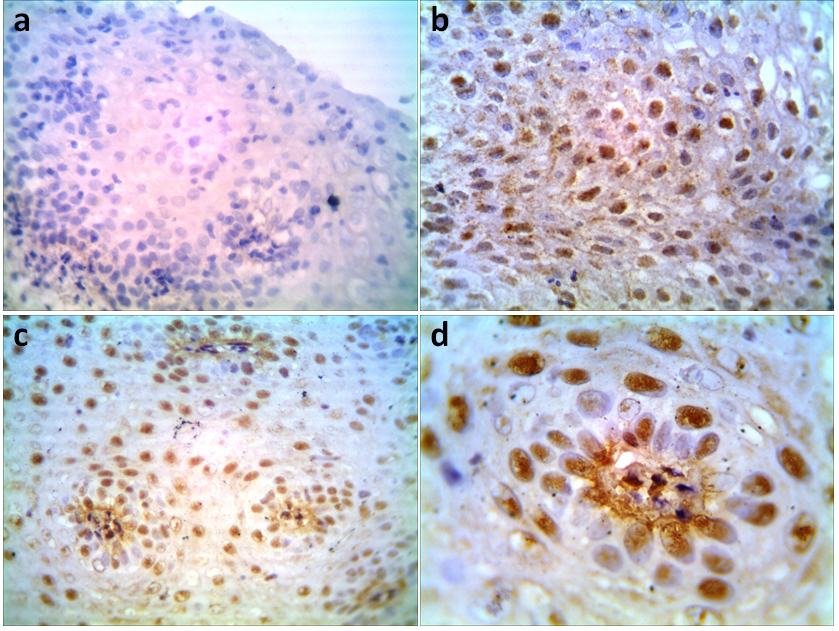

Supplement: Additional file 1 — Figure S1. HR-HPV detection by ISH. Representative images of (a) L-SIL sample in which HPV-probe set was excluded (negative control); (b-d) HR-HPV positive H-SIL showing strong nuclear staining. (a-c) 40×, (d) 100 ×. [file 1471-2407-12-116-S1.TIFF]

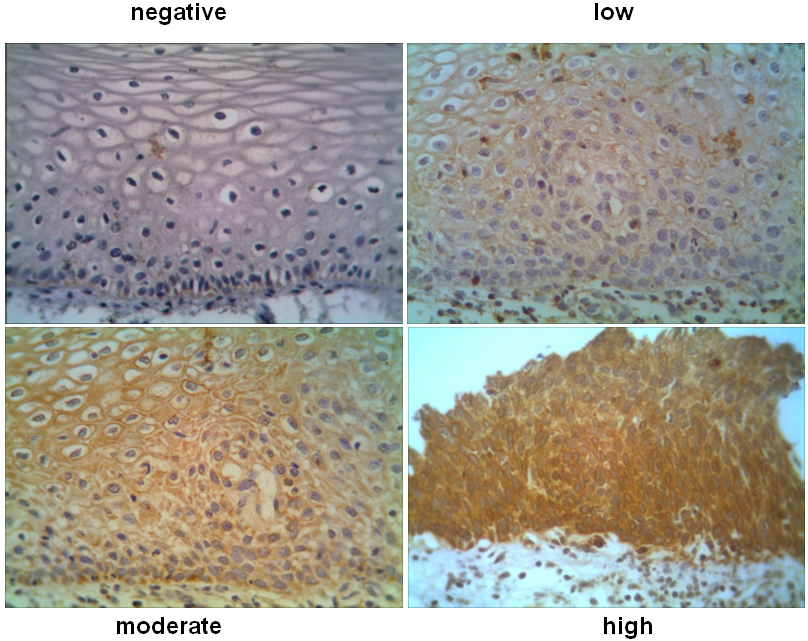

Supplement: Additional file 2 — Figure S2. Establishment of the criteria for interpretation of IHC results. To analyze the differences in the intensity in immunoreactivity of the five proteins, we defined four categories based on signal intensity: a) negative, b) low, c) moderate and d) strong. [file 1471-2407-12-116-S2.TIFF]
