# Supplementary material for: Nuclear expression of Rac1 in cervical premalignant lesions and cervical cancer cells
Source: BMC Cancer. 2012 Mar 23;12:116. doi: 10.1186/1471-2407-12-116 (PMC3340301; doi:10.1186/1471-2407-12-116)
Supplement: Additional file 3 — Table S1. Characteristics of the study population. [file 1471-2407-12-116-S3.PDF]

Supplementary Table 1. Characteristics of the study population

	Histological diagnosis		
	without SIL	L-SIL	H-SIL
	n (%)	n (%)	n (%)
**Age**			
20-30	0 (0)	17 (29.0)	9 (29.0)
31-40	4 (20)	20 (39.2)	14 (45.2)
41-50	11 (55)	11 (21.6)	3 (9.7)
>50	5 (25)	3 (5.9)	5 (16.1)
**HR-HPV**			
negative	10 (50)	8 (15.7)	2 (6.5)
positive	2 (10)	35 (68.3)	25 (80.6)
non-determined	8 (40)	8 (15.7)	4 (12.9)
Total	***20***	***51***	***31***
